# Role of Phytohormones in *Piriformospora indica*-Induced Growth Promotion and Stress Tolerance in Plants: More Questions Than Answers

**DOI:** 10.3389/fmicb.2018.01646

**Published:** 2018-07-31

**Authors:** Le Xu, Chu Wu, Ralf Oelmüller, Wenying Zhang

**Affiliations:** ^1^Hubei Collaborative Innovation Center for Grain Industry, School of Agriculture, Yangtze University, Jingzhou, China; ^2^College of Horticulture and Gardening, Yangtze University, Jingzhou, China; ^3^Matthias-Schleiden-Institute, Plant Physiology, Friedrich-Schiller-University Jena, Jena, Germany

**Keywords:** phytohormones, *Piriformospora indica*, growth promotion, stress tolerance, root–microbe interaction, signal transduction

## Abstract

Phytohormones play vital roles in the growth and development of plants as well as in interactions of plants with microbes such as endophytic fungi. The endophytic root-colonizing fungus *Piriformospora indica* promotes plant growth and performance, increases resistance of colonized plants to pathogens, insects and abiotic stress. Here, we discuss the roles of the phytohormones (auxins, cytokinin, gibberellins, abscisic acid, ethylene, salicylic acid, jasmonates, and brassinosteroids) in the interaction of *P. indica* with higher plant species, and compare available data with those from other (beneficial) microorganisms interacting with roots. Crosstalks between different hormones in balancing the plant responses to microbial signals is an emerging topic in current research. Furthermore, phytohormones play crucial roles in systemic signal propagation as well as interplant communication. *P. indica* interferes with plant hormone synthesis and signaling to stimulate growth, flowering time, differentiation and local and systemic immune responses. Plants adjust their hormone levels in the roots in response to the microbes to control colonization and fungal propagation. The available information on the roles of phytohormones in beneficial root–microbe interactions opens new questions of how *P. indica* manipulates the plant hormone metabolism to promote the benefits for both partners in the symbiosis.

## Introduction

Phytohormones, such as auxins (e.g., IAA, the most common auxin), CK, GAs, ABA, ET, SA, and jasmonates play important roles in growth and development of plants as well as in plant resistance/tolerance to abiotic stresses and in plant interactions with various mutualistic fungi (Cakmak and Marschner, 1992; Sirrenberg et al., 2007; Vadassery et al., 2008; Schäfer et al., 2009a,b; Camehl et al., 2010; Lee et al., 2011; Gutjahr and Parniske, 2013; Johnson et al., 2013; Miransari et al., 2014; Zhang Y. et al., 2015; Kopittke, 2016; Naser and Shani, 2016; Tian et al., 2017; Inahashi et al., 2018; Mathur et al., 2018). Endo- and ectomycorrhizal fungi, N_2_-fixing rhizobacteria and a large number of fungal and bacterial endophytes colonize the roots and confer benefits to the roots in addition to its effects to the leaves. The spectrum of benefits for their host plants includes growth promotion, higher yields, and enhanced resistance/tolerance to several biotic (e.g., pathogens, pests, or nematodes) and abiotic (e.g., drought, salinity and low temperature) stresses. On the other hand, defense-related phytohormones control roots colonization which is essential for stabilizing a symbiotic interaction. *Piriformospora indica* (family Sebacinaceae, order Sebacinales) is a well-known endophytic root-colonizing fungus which was originally discovered in association with xerophytic plants of the Thar Desert in India (Verma et al., 1998). Similar to arbuscular mycorrhizal fungi, *P. indica* promotes growth of the host plants and enhances resistance to microbial pathogens/insects and tolerance to abiotic stress (Harman, 2011). In the past decade, more information has become available regarding the roles of phytohormones in fungus–plant interactions. Growth and yield benefits are largely attributed to the production of the phytohormones IAA, CK, and GA, whereas enhanced stress tolerance is often explained by a systemic resistance response involving ABA, ET, SA, and jasmonates, and their interactions with *P. indica* have been reported recently (Varma et al., 2012; Gill et al., 2016). However, none of these hormones can be considered individually, since there is an enormous crosstalk between them. The crosstalk between JA, SA, (and ET) is long known (cf. Caarls et al., 2015; Yang et al., 2015; Zhang P.J. et al., 2015); only recently, it became clear that two ABA-responsive plastid lipases are involved in JA biosynthesis (Avramova, 2017; Wang et al., 2018). Many other processes in symbiotic interactions of roots with microbes require intensive crosstalk between phytohormones (e.g., Pozo et al., 2015; Gamas et al., 2017; Hu et al., 2017; Liu et al., 2017; Tognetti et al., 2017; Shen et al., 2018, to mention a few). Here, we discuss the importance of phytohormones and their crosstalks in the interaction of *P. indica* with a wide range of higher plants. Based on studies of beneficial root symbioses and pathosystems, we highlight open questions which can be experimentally addressed with an endophyte like *P. indica* during its interaction with model plants and agriculturally important crops.

## Auxin

Auxins (such as IAA, the most abundant auxin in nature) coordinate many key processes in plant development and adaptive growth (Naser and Shani, 2016), and therefore, it is not surprising that root-colonizing microbes including *P. indica* interfere with the plant auxin metabolism or signaling for stimulating growth and developmental processes in their hosts. Besides the involvement of auxin in cell division, cell enlargement and organ development, it also participates in defense and stress responses (Kopittke, 2016; Egamberdieva et al., 2017), which makes this hormone an interesting target for root-interacting microbes. How *P*. *indica* targets auxin to the responsive cells in the roots, and how it establishes new and/or controls auxin maxima have not yet been investigated. It is long known that many root-colonizing beneficial and pathogenic bacteria produce IAA (e.g., *Agrobacterium tumefaciens*, *A. rhizogenes*, *Pseudomonas syringae* and other sp., *Erwinia herbicola*, species of *Azospirillum*, *Azotobacter*, *Rhizobium*, *Bacillus*, and *Enterobacter* (Glick, 1995; Patten and Glick, 2002; Ouzari et al., 2008; Abd El-Hadi Nadia et al., 2009; Khan and Doty, 2009; Ahmed and Hasnain, 2010; Merzaeva and Shirokikh, 2010; Roy et al., 2010; Fu and Wang, 2011; Patil, 2011; Celloto et al., 2012). Also root-colonizing beneficial and pathogenic fungi produce auxins (Reineke et al., 2008; Felten et al., 2009, 2010; Splivallo et al., 2009; Fu et al., 2011; Robert-Seilaniantz et al., 2011; Sukumar et al., 2013; Fusconi, 2014; Gutjahr, 2014; Raudaskoski and Kothe, 2015). For instance, the IAA producing *Metarhizium robertsii* promotes lateral root growth and root hair development in *Arabidopsis* and auxin is involved in enhancing virulence to insects (Liao et al., 2017). *M. robertsii*-colonized plants withstand attacks by herbivores in part by emitting volatiles that attract insect predators and bolster resistance to future threats. IAA is particularly important in regulating plant defensive responses and it is likely to accumulate at the site of insect feeding (Peck and Kende, 1998; Howe and Jander, 2008), which could make it a useful host-related signal triggering infection-related processes in *M. robertsii*. However, the exact mechanisms remain to be confirmed. Also auxin transport can be affected by beneficial microbes (Felten et al., 2009; Ng et al., 2015). The hormone may quantitatively influence AM colonization and perform an additional cell-autonomous function in the promotion of arbuscule development (Gutjahr, 2014). Several reports demonstrate that *P. indica* interferes with auxin production and signaling in the hosts and is responsible for, or at least contribute to, root growth (Sirrenberg et al., 2007; Vadassery et al., 2008; Lee et al., 2011; Hilbert et al., 2012, 2013; Dong et al., 2013; Ye et al., 2014; Kao et al., 2016; Hua et al., 2017). Sirrenberg et al. (2007) first demonstrated that *P. indica* produced IAA in liquid culture and that fungal auxin production affects root growth. The auxin levels vary substantially in different plant species colonized by *P. indica*, which has been measured either directly or deduced from the expression of auxin-responsive genes. For example, large-scale microarray analysis of *P. indica*-colonized *Arabidopsis* roots showed that auxin-related gene expression and the auxin content remained unchanged in the entire root in response to *P. indica* (Vadassery et al., 2008). Lee et al. (2011) showed that growth promotion of Chinese cabbage (*Brassica campestris*) and *Arabidopsis* by *P. indica* is not stimulated by mycelium-synthesized auxin. Also exogenous application of auxins to *Arabidopsis* seedlings could not mimic the growth promotion effect induced by the fungus. However, *P. indica* rescued the dwarf phenotype of the *Arabidopsis* auxin overproducer *sur1-1* by converting free auxin into conjugates, resulting in down-regulation of the auxin-induced *IAA6* gene (Vadassery et al., 2008). Apparently, high concentrations of active auxin, which inhibit growth in the roots and aerial parts of plants, are negated by the presence of *P. indica*. Additionally, *Arabidopsis* mutants with reduced auxin levels exhibit a normal colonization response to *P. indica*, suggesting that *P. indica*-promoted *Arabidopsis* growth results from alterations in auxin homeostasis (Vadassery et al., 2008). Promotion of growth and development in *P. indica*-colonized Chinese cabbage and barley seedlings have been attributed to increased levels of auxin in roots, while the auxin level in leaves remained unaffected (Lee et al., 2011; Hilbert et al., 2012). Auxin transport-related and signaling proteins such as AUX1 and PINs in Chinese cabbage were up-regulated at the mRNA level by *P. indica* inoculation, suggesting a positive role of the fungus on the plant auxin metabolism (Lee et al., 2011). These analyses are based on hormone levels in entire organs (roots or shoots), and local auxin maxima which are required for root growth in the maturation zone or for the initiation of lateral root formation were not considered in these studies.

In Chinese cabbage roots, *P. indica* mainly stimulated growth promotion in the maturation zone and this involves the up-regulation of auxin-responsive genes (Dong et al., 2013). By using high-throughput gas-chromatography-based mass spectrometry, Hua et al. (2017) demonstrated that auxin and its intermediates were induced and *de novo* synthesized in colonized Chinese cabbage roots. Ye et al. (2014) performed a genome-wide expression profiling of small RNAs in *Oncidium* orchid roots either colonized or not-colonized by *P. indica*. The predicted target genes of these miRNAs are mainly involved in auxin signal perception and transduction, transcription, development and plant defense. The mRNA level for the auxin receptor protein TIR1 showed a gradual downregulation within the first 8 weeks after *P*. *indica* inoculation of Oncidium orchid roots, while the mRNAs for auxin response factors (ARFs) were up-regulated during the first week followed by down-regulation during later phases of the symbiosis (Ye et al., 2014). Although these results clearly demonstrate the involvement of auxin in the symbiotic interaction of various plant species with *P. indica*, the source of the phytohormone and how it is generated and active remained unclear, until Hilbert et al. (2012) showed that indole derivative production by *P. indica* is not required for growth promotion but for biotrophic colonization of barley roots. They showed that *P. indica* produce IAA, but also indole-3-lactate (ILA) which is synthesized through the intermediate indole-3-pyruvic acid (IPA). Time course transcriptional analyses after exposure to tryptophan designated the *P. indica piTam1* gene as a key player. A green fluorescence protein reporter study and transcriptional analysis of colonized barley roots showed that *piTam1* is induced during the biotrophic phase. *P. indica* strains in which the *piTam1* gene was silenced *via* an RNA interference approach were compromised in IAA and ILA production and displayed reduced colonization of barley roots in the biotrophic phase, but the elicitation of growth promotion was not affected compared with the wild-type situation. Their results suggest that IAA is involved in the establishment of biotrophy in *P. indica*-barley symbiosis and might represent a compatibility factor in this system (Hilbert et al., 2012).

Many soil-borne fungi directly target the plant root system and auxin production, as also shown for lateral root production in mycorrhized maize (Kaldorf et al., 2005). In soybean (*Glycine max*), exogenous auxin induces phosphate uptake, demonstrating that plant growth promotion by *P. indica* might also be the result of improved soil exploitation achieved by auxin-induced root branching (Shen et al., 2006). The bushy root hair phenotype of Chinese cabbage roots conferred by the enhanced auxin content resulting from *P. indica* colonization promotes acquisition of water and minerals (Lee et al., 2011). The described differences of *P. indica* in auxin levels and functions in different plant species might be caused by different sensitivities of the species to growth stimulating signals. For example, the Chinese cabbage cultivars used for the co-cultivation analyses (which are mainly used for vegetable production) are optimized to produce large biomasses and might therefore be more susceptible to growth stimulating signals than *Arabidopsis* which was not under such a selection pressure.

The addition, low concentrations of IAA in barley also led also to suppression of the oxidative burst (Hilbert et al., 2012), suggesting that the IAA produced by the fungus also interferes with host plant defense. The involvement of auxin in balancing growth and defense responses has been often postulated (cf. Egamberdieva et al., 2017). Stringlis et al. (2017) showed that *Pseudomonas simiae* WCS417 promotes *Arabidopsis* growth and induces systemic resistance without being warded off by local root immune responses. Using the auxin response mutant *tir1afb2afb3*, they demonstrated a dual role for auxin signaling in finely balancing growth-promoting and defense eliciting activities of beneficial microbes in plant roots. This requires cross-talks between auxin and defense-related phytohormones. Considering the central role of auxin in beneficial root–microbe interactions, it will be an important task to understand how the symbionts manage to regulate the trait-off between growth and defense in their hosts. Future studies should focus on specific tissues or cells which are targeted by beneficial fungi, and how biologically active auxin maxima are established in response to root colonization, especially, the roles of small secreted proteins from *P. indica* in auxin synthesis and signaling in the specific tissues or cells. Combinations of –omics approaches, phytohormone analyses and tissue/cell-specific responses in wild-type and auxin mutants will help to unravel the complex roles of auxin in the multiple responses elicited by the beneficial fungi. Studies on the role of auxin in pathogenic plant/microbe organisms (Kunkel and Harper, 2018) or under stress (Kazan, 2013; Naser and Shani, 2016) will provide us with additional tools to understand how auxin is involved in balancing beneficial and non-beneficial traits in symbiotic interactions.

## Cytokinin (Ck)

Although CKs are involved in many basic processes including photosynthesis and growth regulation, maintenance of cell proliferation, cell differentiation and retardation of senescence, their role in symbiotic interactions is less clear (Boivin et al., 2016). Besides regulation of the plant CK level, metabolism and signaling in response to root-colonizing microbes, CKs are also produced by rhizospheral microorganisms themselves (Podlešáková et al., 2013; Fusconi, 2014; Kudoyarova et al., 2014). Furthermore, CKs interact with other phytohormones or control defense processes in symbiotic roots (e.g., Bishopp et al., 2011; Jiang et al., 2013). CKs travel from shoot to root and *vice versa* thereby distributing information within the entire plant. Shoot CK has a positive impact on arbuscular mycorrhizal fungal development in roots and on the root transcript level of the AM-responsive phosphate transporter gene *NtPT4* in tobacco. Reduced CK content in roots caused shoot and root growth depression of AM-colonized plants (Cosme et al., 2016b). Systemic shoot-to-root signaling also negatively regulated nodulation in legumes (Sasaki et al., 2014). Moreover, symbiotic root-to-shoot information transfer is necessary for the benefits in the aerial parts of root-colonized plants. Ko et al. (2014) provide molecular evidence for the long-distance transport of CK and showed that AtABCG14, an ABC transporter, is crucial for the translocation of CK from the roots to the shoot, and this is required for normal shoot development. *Trans*-zeatin-riboside, a CK precursor, is a major long-distance root-to-shoot signaling molecule in xylem vessels and its action depends on metabolic conversion via the LONELY GUY enzyme in proximity to the site of action. An additional long-distance signaling molecule is *trans*-zeatin, an active CK form. *Trans*-zeatin, a minor component of xylem CK, controls leaf size but not meristem activity-related traits, whereas *trans*-zeatin riboside is sufficient for regulating both traits (Osugi et al., 2017, and ref. therein). These observations raise the question whether CKs are involved in microbe-induced systemic signaling, and – if so – how this is related to other components which also distribute information within the plant body.

The information on the involvement of CK in the colonization of the roots by *P. indica* as well as systemic signal transfer is mainly descriptive. *P. indica* targeted CK-responsive genes in local root tissues and systemic leaves of colonized plants have been identified in several plant species (e.g., Vadassery et al., 2008; Johnson et al., 2014; Zhang et al., 2018). Vadassery et al. (2008) demonstrated that *trans*-zeatin biosynthesis and the receptor combination CERK1/AHK2 are important for *P. indica*-mediated growth promotion in *Arabidopsis* seedlings, while mutants lacking *cis*-zeatin showed a wild-type response to the fungus. Since *trans*-zeatin and its precursor controls the leaf size but not meristem activity-related traits whereas *trans*-zeatin riboside is sufficient to control both traits (Osugi et al., 2017) via systemic root-to-shoot signaling, it is conceivable that the information transfer from colonized roots to the leaves requires or is dependent on *trans*-zeatin.

Besides their involvement in systemic signaling, CKs play different roles in the entry of mirco-symbionts into the cortex: they inhibit entry of rhizobacteria and promote colonization of mycorrhizal fungi (Jones et al., 2015). A similar function has been attributed to ABA (López-Ráez, 2016). It would be interesting to understand whether CKs have the same or similar function on root colonization or hyphal entry into the root cell by endophytes, such as *P. indica*.

A quite interesting study by Hérivaux et al. (2017) showed that histidine kinases in early diverging fungi share a high degree of similarity with phytohormone receptors including those for CK and ET perception. These phytohormone receptor homologs are found in plant root symbionts but also in species that colonize decaying plant material. Hérivaux et al. (2017) hypothesize that these sensing proteins promote the fungal interaction with plants, which might have led to the conquest of land by ancestral fungi. The hypothesis that plant-derived CKs might be perceived by fungal perception systems, is appealing considering that *P. indica* shows similar symbiotic and saprophytic features as the diverging fungi analyzed early.

Furthermore, there is increasing evidence that volatiles from microbes in the rhizosphere contribute to the performance of plants in ecosystems. Sánchez-López et al. (2016) showed that volatiles emitted by phylogenetically diverse rhizosphere and non-rhizosphere bacteria and fungi (both pathogens and beneficial microbes) promote growth and flowering of various plant species, including crops and *Arabidopsis*. CKs play essential roles in this phenomenon, because growth and flowering responses to the volatiles were reduced in mutants with CK-deficiency (e.g., *35S*:*AtCKX1*) or low receptor sensitivity (e.g., *ahk2/3* mutants). They suggested that plants react to microbial volatiles through highly conserved regulatory mechanisms. Although the role of volatiles in the interaction of *P. indica* with various plant species is not well understood, their potential involvement in growth responses can be analyzed in the CK mutants.

Taken together, CK may participate in *P. indica*-induced physiological responses in host plants which have not yet been analyzed or for which an involvement of CK has not yet been investigated.

## Gibberellin (GA)

Gibberellin has become a novel player in orchestrating symbiotic interactions of plants with microorganisms (Foo et al., 2014). Many investigations demonstrated that GAs crosstalk with other phytohormones and are involved in stress resistance, in particular salt stress (reviewed in Egamberdieva et al., 2017). DELLA proteins are master negative regulators of GA signaling, and integrate quite diverse hormone signaling pathways and pathways from developmental processes. They also control and coordinate many steps in root development, including the formation of endosymbiotic interactions with rhizobial bacteria and mycorrhizal fungi (Yu et al., 2014; Fonouni-Farde et al., 2016). GA signaling inhibits AM formation (Gutjahr, 2014; Martín-Rodríguez et al., 2015, cf. also Ortu et al., 2012; Takeda et al., 2015a,b), and GA is controlled by ABA (Martín-Rodríguez et al., 2016). *Reduced Arbuscular Mycorrhiza1* (RAM1), a GRAS protein, regulates arbuscule development, and a *Lotus japonicas* mutant impaired in RAM1 function is arrested in arbuscule branching. Symbiotic and GA signals control RAM1 expression and arbuscule development (Pimprikar et al., 2016). In pea roots, AM colonization was increased in GA-deficient mutants while a mutant which lacks DELLA proteins showed reduced colonization (Foo et al., 2013, 2014, 2016).

In contrast, quite little is known about the role of GA in interactions of endophytes with roots, and the available studies on *P. indica* mainly focused on the activation of innate immune responses. Cosme et al. (2016a) showed that *P. indica* helps rice plants to tolerate root herbivory, and GA functions as a signal component of inducible plant tolerance against the biotic stress. Barley mutants impaired in GA synthesis as well as perception showed reduced colonization by *P. indica* which implicates that GA functions as a modulator of the root’s basal defense (Schäfer et al., 2009b). A quintuple-DELLA mutant displayed constitutive GA responses and the GA biosynthesis mutant *ga1-6* (for *GA requiring 1*) showed higher and lower degrees of colonization in *Arabidopsis* roots, respectively, suggesting that *P. indica* recruits GA signaling to establish root cell colonization (Jacobs et al., 2011). Ent-kaurene synthases and ent-kaurene-like synthases are involved in the biosynthesis of phytoalexins and/or GAs. Li et al. (2016) demonstrated that kaurene synthase activity is required for successful root colonization of *P. indica* in barley. Finally, *P. indica*-induced growth promotion of Chinese cabbage and barley seedlings correlated to an increased GA level in the colonized roots (Schäfer et al., 2009b; Lee et al., 2011), and the *GA2ox* gene involved in inactivation of GA was down-regulated in *P. indica*-colonized barley roots (Hedden, 2008; Schäfer et al., 2009b). In most of these publications, a crosstalk to or involvement in other phytohormones is proposed to induce the described effects. Whether all these observations are based on a common GA mechanism, requires more investigations.

Gibberellin is involved in early flowering phenotype, and *P. indica* promotes early flowering and plant growth in the medicinal plant *Coleus forskohlii* and *Arabidopsis*. To determine the impact of *P. indica* on flowering time in *Arabidopsis*, Kim et al. (2017) co-cultivated *Arabidopsis* plants with the fungus under long day condition, and these plants displayed an early flowering phenotype. The same results were reported by Pan et al. (2017). Colonized plants had higher transcript levels of the flowering regulatory genes *FLOWERING LOCUS T*, *LEAFY*, and *APETALA1*, as well as of the GA biosynthetic genes *Gibberellin 20-Oxidase 2*, *Gibberellin 3-Oxidase 1* and *Gibberellin Requiring 1*, while the flowering-repressing gene *FLOWERING LOCUS C* was down-regulated. Quantification of GA contents showed that the colonization by *P. indica* caused an increase in GA_4_ content. Compared to wild-type, inoculation of the *Arabidopsis ga5* mutant defective in an GA biosynthetic gene led to less pronounced changes in the expression of genes regulating flowering and to a lower increase in GA_4_ content. Taken together, the two studies demonstrated that *P. indica* promotes early flowering in *Arabidopsis* likely by increasing the GA content.

## Abscisic Acid (ABA)

Abscisic acid is a classical stress hormone and involved in many osmotic responses including salt and drought stress. A classical ABA response is the closure of the stomata, which prevents or restricts water loss and pathogen entry into the host plants. ABA often represses plant immune responses, and pathogens also utilize or synthesize ABA for suppression or manipulation of the immunity of their hosts (Mbengue et al., 2016; Lievens et al., 2017). The importance of ABA for beneficial symbiotic interactions has recently become an interesting research field (Lievens et al., 2017). In general, ABA promotes AM symbiosis although the effect of the hormone is strongly dependent on the developmental stage of the interaction and stress conditions (López-Ráez, 2016). In contrast, its role in the legume–rhizobia interaction is mainly inhibitory (Lievens et al., 2017). ABA comes into play when plants and their associated symbionts are exposed to stresses, in particular osmotic stress. Unstressed plants with sufficient nutrient supply do not require help from symbionts. Vahabi et al. (2015) showed that the benefits for the plants in the symbiotic interaction between *P. indica* and *Arabidopsis* increase with increasing moderate stress, because the plants have to decide whether they invest in either stress tolerance responses or symbiotic features. Stec et al. (2016) proposed that the fluctuation in the ABA levels may work as an alert system that calculates the ratio between costs and incomes. The ABA level may function as a monitor for the decision whether an investment in a particular symbiotic interaction is higher than the profit coming out of it. In the light of such a hypothesis, ABA regulation in response to *P. indica* colonization might be interesting since the growth and stress conditions can be experimentally manipulated.

A strong up-regulation of the ABA concentration during *P. indica* colonization was observed in *Arabidopsis* roots and shoots during the early recognition phase and before a physical contact between the two symbiotic partners (Vahabi et al., 2015). Apparently, mobile signaling molecules from the fungus, such as cellotriose (Johnson et al., 2018) or small secreted proteins (Akum et al., 2015), inform the plants about the presence of the microbe, and activate ABA-dependent stress responses. The plants also close the stomata. Interestingly, the ABA levels decreased in both roots and shoots when a physical contact between the two organisms was established (Vahabi et al., 2015). It appears that the microbe is no longer recognized as a threat. ABA is also involved in plant innate immune responses, and may thereby interfere with or even control root colonization. Peskan-Berghöfer et al. (2015) showed that treatment of *Arabidopsis* seedlings with exogenous ABA or the ABA analog pyrabactin increased fungal colonization efficiency without impairment of plant fitness. Concomitantly, the ABA-deficient mutants *aba1* and *aba2* were less colonized, while plants exposed to moderate stress were more colonized than corresponding controls. Sustained exposure to ABA attenuated expression of transcription factors MYB51, MYB122, and WRKY33 in roots upon *P. indica* challenge. Thus, ABA strengthens the interaction of *Arabidopsis* roots with *P. indica* as a consequence of its impact on plant innate immunity. This will also have a strong impact on the establishment and outcome of the symbiosis under stress conditions. Similar results have been obtained with arbuscular mycorrhizal fungi, where ABA promotes the infection process and establishment of the compatible interaction (Herrera-Medina et al., 2007; Charpentier et al., 2014; Fracetto et al., 2017; Lievens et al., 2017). Salomon et al. (2014) also demonstrated that root-colonizing bacteria were able to interfere with their host plants by producing ABA. In summary, the available data support a concept in which ABA plays an important role in the control of beneficial traits in the symbiosis, in particular under stress, and may even function as sensor.

## Ethylene (ET)

The plant hormone ET controls flowering, ripening, seed dormancy, seed germination, senescence, root formation, plant growth, and adaptation under abiotic and biotic stress (Kende, 2003; Abeles et al., 2012). The gaseous hormone is synthesized from the precursor ACC, which derives from methionine and ACC synthase and ACC oxidase are key enzymes in this biosynthetic pathway.

The role of ET in mutualistic interactions is complicated and not clear, and numerous reports demonstrated its involvement in quite different steps. Correlations of ET emissions with *P. indica*-induced responses in the hosts have not yet been performed and the participation of this hormone in *P. indica*–host interactions is mainly based on mutant analyses or altered expression of ET-responsive genes. Some plant-growth promoting bacteria, such as *Rhizobacteria* spp., degrade the ET precursor ACC with an ACC deaminase, which presumably promotes growth of the microbes by repressing hosts’ defense system which is activated by ET (cf. Shaharoona et al., 2006). A gene encoding such an enzyme has not been found in the *P. indica* genome. In many studies, colonization of roots by mycorrhizal fungi or rhizobacteria is inhibited by ET, however, there are also reports showing opposite effects (cf. Khatabi and Schäfer, 2012). In the root–*P. indica* interaction, the fungus positively modulates expression of ACC synthase (Khatabi et al., 2012; Ansari et al., 2013), and ET has been implicated as a positive modulator of the symbiosis in *Arabidopsis* and barley roots (Khatabi et al., 2012). Moreover, DNA microarray-based gene expression analysis of barley roots colonized by *P. indica* showed differential expression of genes related to ET synthesis and signaling (Khatabi et al., 2012). *P. indica* induces ET synthesis in barley and *Arabidopsis* during colonization and *Arabidopsis* mutants impaired in ET signaling were less colonized by the fungus and mutants with constitutive ET signaling, ET synthesis or ET-related defenses were hyper-susceptible to *P. indica*. The fungus also induces methionine synthase which may provide more substrates for ET synthesis (Peskan-Berghöfer et al., 2004). The observation that ET supports root colonization is consistent with the requirement of JA for root colonization (cf. below, Verma et al., 1998), and the two hormones function synergistically in the host in response to *P. indica* colonization. ET induces innate immune responses against pathogenic microbes, but also mildly in response to beneficial root-colonizing fungi, for example before recognition of the microbe as a friend, for restriction of root colonization or secondary colonization of distal root parts by the same microorganism. Thus, ET is probably one of many factors which plays role in balancing beneficial and non-beneficial colonization traits through signaling components in *P. indica* symbiosis (Camehl et al., 2010).

The growth of *Arabidopsis* mutants impaired in ET biosynthesis and activation of ET response (*etr1*, *ein2, and ein3/eil1*), were not promoted or even inhibited by *P. indica* (Camehl et al., 2010), suggesting that these ET signaling components are required for *P. indica*-induced growth promotion in *Arabidopsis*. In older plants, which are already colonized by the fungus, *ETR1*, *EIN2*, and *EIN3*/*EIL1* participate in restricting root colonization and repressing defense responses. Consequently, ET perception and signaling, as well as ET-targeted transcription factors, are crucial for *P. indica*-induced growth promotion in *Arabidopsis* (Camehl et al., 2010). The available data suggest that ET might have different effects during different stages of the symbiosis in balancing beneficial and non-beneficial traits in the symbiosis. This is not surprising considering the huge amount of ET targets and ET-targeted transcription factors. More than 100 ET RESPONSE FACTORS (ERFs) affect quite different downstream responses both positively and negatively ranging from development, metabolic processes to defense gene activation. Camehl and Oelmüller (2010) showed that two ERFs, ERF9 and ERF14, are required for the growth promoting response of *P. indica* in *Arabidopsis*. Finally, *P. indica* confers salt-stress tolerance in barley (Ghaffari et al., 2016). The fungus-induced reprogramming during salt stress affects major metabolic and transcriptomic processes including the ET biosynthesis pathway. The involvement of the gaseous hormone in major developmental steps and its crosstalk with other hormones makes it difficult to define specific effects in the stabilization of a beneficial symbiosis under changing environmental and developmental conditions. ET also participates in rhizobacteria-induced induced systemic resistance (ISR), where the microbes activate processes in roots which induce systemic resistance in the aerial parts of the plants against microbial pathogens or insects (Verhagen et al., 2004). *P. indica* has been shown to induce ISR in various plant species, and ET and jasmonates have been proposed to be involved in the signal transfer and/or realization (cf. Molitor and Kogel, 2009). ET as a gaseous hormone, can easily stimulate these responses systemically in distant tissues, but also ACC travels from the roots to the aerial parts via the xylem (Bradford and Yang, 1980; Finlayson et al., 1991; Tudela and Primo-Millo, 1992, and ref. therein). Finally, ET might also play a role in interplant communication, by informing adjacent plants about upcoming threat in a plant community. Understanding of the role of ET in plant–microbe interaction requires probably broader experimental approaches which should consider also other players and hormones in the communication networks.

A recent study by de Zélicourt et al. (2018) shows a novel role of ET in stress tolerance. The desert endophyte *Enterobacter* sp. SA187 enhances yield of the crop plant alfalfa under field conditions as well as growth of *Arabidopsis in vitro*. Among the different mechanisms related to the beneficial association of SA187 with plants, the bacterium induces salt stress tolerance by production of 2-keto-4-methylthiobutyric acid (KMBA) which is known to be converted to ET. Using this novel chemical compounds, the authors demonstrated that the *Arabidopsis* ET signaling pathway, but not ET production is required for KMBA-induced plant salt stress tolerance. Since ET has been proposed to be involved in numerous plant resistance responses in symbiotic interactions, it remains to be seen, whether the novel chemical mediator KMBA is also produced by other endophytes.

## SA and JA, Two Phytohormones Involved in Quite Different Defense Strategies

The two phytohormones SA and JA mediate defense responses and systemic signal propagation in plant–microbe interactions, whereas SA is mainly involved in the response to biotrophic microbes and JA (and ET) to necrotrophic microbes. Studies have also revealed the dependency of *P. indica* on JA-mediated suppression of early immune responses (e.g., root oxidative burst) as well as SA- and glucosinolate-related defense pathways (Jacobs et al., 2011; Gill et al., 2016). To ensure that plants establish a proper response to attacking pathogens, there is a massive crosstalk between the two hormone signaling pathways, in which one hormone represses the action of the other. In many aspects, initial steps in establishing beneficial plant–microbe interactions resemble those of biotrophic interactions, therefore, up-regulation of SA levels and intensified SA signaling is likely, in particular since SA is also known to participate in ISR responses, which are often induced by root-colonizing beneficial microbes. However, this appears to be too simplified considering the literature published on *P. indica*–host interactions.

Mutualistic interactions are characterized by several phases: during early phases of recognition, the host has not yet physically contacted the microbe and recognized it as a friend. This results in an activation of a mild defense response which involves SA and/or jasmonates. Vahabi et al. (2015) showed that both SA and jasmonate levels increased when *Arabidopsis* roots were growing next to *P. indica* before a physical contact has been established. This suggests that the growing hyphae release chemical mediators which activate SA and jasmonate biosynthesis genes in the roots. During a second phase, the two symbionts have physical contact and root colonization is initiated, which results in a local repression of the host’s defense response and lower SA and jasmonate levels (Vahabi et al., 2015). This can be caused by numerous factors: effector proteins released by the microbes can actively repress plant immune responses, but progression of root colonization can also “convince” the plant that the microbe is friendly. Down-regulation of the defense machinery in the host may be a consequence of the beginning nutrient exchange. Ultimately, a balance between growth of the microbes in the host and the profit of both symbiotic partners from the symbiosis has to be established, and this balance requires plant defense strategies to restrict the progression of root colonization. Analyses of phytohormone mutants demonstrated that SA, JA, and ET are crucial players in establishing and maintaining the balance, and the participation of the individual hormones depends on the developmental stage and growth conditions. Apparently, both SA and jasmonates are involved in controlling root colonization at the infected sites and the colonization success essentially depends on the evolution of strategies for immunosuppression (Jacobs et al., 2011).

In contrast, when *Arabidopsis* roots are directly exposed to *P. indica* hyphae, the JA and JA-Ile levels are up-regulated, although the benefits for the plants were not lost (Vahabi et al., 2013). Apparently, under these conditions, the microbe is recognized as threat. Similarly, Lahrmann et al. (2015) found that *P. indica* as well as *Sebacina vermifera* stimulated the SA catabolism and the accumulation of jasmonates and glucosinolates in *Arabidopsis* roots. In the context of the saprophytic traits of the microbes they concluded that the symbiotic interaction of these microbes requires non-compromised plant innate immune responses. Vahabi et al. (2016) investigated beneficial and non-beneficial traits in the symbiotic interaction between *P. indica* and *Arabidopsis* roots and found that mild stress (limited access to nutrients, exposure to heavy metals or salt, light and osmotic stress, pathogen infections) promotes the benefits for the plant in the symbiosis. A physical contact was necessary, suggesting that the endophyte helps the plant after recognition of *P. indica* as a friend. Furthermore, the level of active jasmonates increased under increasing stress conditions. A testable hypothesis could be that shifting the symbiosis from beneficial to less beneficial conditions might be associated with a shift from SA to jasmonate usage as the defense-regulating plant hormone.

Akum et al. (2015) investigated the *P. indica* effector protein PIIN_08944, which promotes the mutualistic symbiosis and root colonization. When ectopically expressed in *Arabidopsis* and barley, the effector protein contributed to root colonization by interfering with the SA-mediated basal immune response. Interestingly, the transgenic *Arabidopsis* plants supported the growth of a biotrophic oomycete while growth of necrotrophic fungi on *Arabidopsis* or barley was not affected. This result supports the important role of SA in root colonization and shows that the microbe has specific strategies to interfere with the plant SA pathway.

*P. indica* controls secondary colonization in distal root areas by stimulating JA-dependent defense responses, which demonstrates systemic signaling. While ISR is normally considered as SA dependent, Stein et al. (2008) demonstrated that JA signaling and the cytoplasmic, but not nuclear localization of NPR1 are required for *P. indica*-induced resistance against powdery mildew infection. Two jasmonate signaling mutants were non-responsive to *P. indica* and *JA-responsive vegetative storage protein* expression was primed in response to the pathogen infection. The resistant phenotype was independent of SA and SA biosynthesis and signaling (Molitor and Kogel, 2009). How the information travels to the aerial parts is not known, but several reports suggested that, like in roots, altered phytohormone levels in the leaves suppress host immunity (cf. Jacobs et al., 2011) or prime the aerial parts for better protection against pathogen attack (e.g., Sun et al., 2014). Stimulation of jasmonates and JA-responsive genes by *A. brassicae* infection was strongly inhibited when the plants are colonized by *P. indica* (Vahabi et al., 2013, 2018), suggesting that the colonized plants suppress the host immunity because these plants are stronger and healthier and require less JA-dependent defense for protection. In maize seedlings, the drought stress responses were also diminished by *P. indica*, but in this study, the fungus stimulated the expression of SA-related genes in roots (Zhang et al., 2018). Altered phytohormone levels can also result in a secondary effect: e.g., *P. indica* stimulated the expression of a tau-type glutathione *S*-transferase in Chinese cabbage. When the gene encoding the enzyme was expressed in *Arabidopsis*, the plants performed better and stimulated the expression of auxin-, SA- and JA-responsive genes (Kao et al., 2016). Thus, the fungus-induced reprogramming of the host’s gene expression might alter phytohormone levels.

## Brassinosteroids (BRs)

Other phytohormones synthesized or manipulated by the root endophytes include brassinosteroids (BRs). Direct measurements of these steroid hormones in response to root colonization by *P. indica* have not yet been performed, however, the gene encoding cycloartenol synthase, which contributes to the synthesis of BR precursors, and *BLE2* encoding a BR-responsive nine-transmembrane protein, are up-regulated after *P. indica* inoculation. In addition, two *BAK1* (brassinosteroid insensitive 1-associated receptor kinase 1) genes involved in BR signaling are induced (Schäfer et al., 2009a,b).

## Conclusion

**FIGURE 1:**
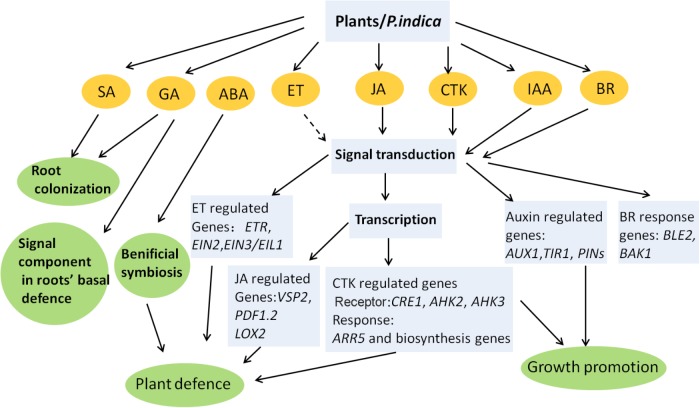
Phytohormone-related processes influenced by *P. indica* in plants. Most of the conclusions are obtained from *Arabidopsis*, barley, and Chinese cabbage. Dashed line represents undefined role. Interactions between different phytohormones are not indicated. The model is adapted from: Gill et al. (2016) and Varma et al. (2012) and enriched by adding new findings.

The symbiotic relationship with *P. indica* promotes growth and enhances stress tolerance in plants, which involves two possible mechanisms. (1) The first is stimulating the action of host stress-responsive systems under a particular stress, which directs the plants to either keep away from or mitigate the stress. The mechanism of drought and salt tolerance mediated by fungi in plants involves the action of CK (Miller, 1974), ABA (Nishiyama et al., 2011), and fungal ACC deaminase to utilize ACC (Viterbo et al., 2010). Additionally, SA and JA are likely involved in ISR conferred by *P. indica* inoculation. (2) Another possible mechanism involves the synthesis of anti-stress biochemicals by endophytes (Singh et al., 2011). Some microbes clearly synthesize auxins or CK, which influence plant metabolism. Fungi also stimulate lateral root formation and increase the root surface area by producing auxins and GA, which help plants take up more water and minerals during stresses, thereby enhancing survival and yield. The role of phytohormones in *P. indica*–plants interactions has been simply summarized in **Figure 1**. In recent years melatonin has emerged as a research highlight in plant studies. Melatonin plays important roles in plant growth and development (Chen et al., 2009), and is related to abiotic stresses such as drought, radiation, extreme temperature, and other harsh environmental conditions (Zhang et al., 2012; Byeon and Back, 2014; Wang et al., 2014; Li et al., 2017). Exposure of plants to environmental stress can increase the level of endogenous melatonin (Zhao et al., 2013). Overexpression of the melatonin biosynthetic genes elevates melatonin levels and enhanced tolerance to abiotic stresses (Zhang et al., 2012). More importantly, the antioxidant system is one of the targets of melatonin in plants stress tolerance, and preliminary data suggest that the melatonin targets are similar to those of root-colonizing microorganisms including *P. indica* (Rodriguez et al., 2010; Narayan et al., 2017). Though melatonin distinguishes from a classic hormone such as its direct, non-receptor-mediated free radical scavenging activity, it could cross talk with stress or toher hormones, like IAA, ABA, JA, SA, and ET (Zhang N. et al., 2015). How the beneficial effects induced by *P. indica* are related to melatonin needs to be elucidated.

Thus, the fungus is ideal to study many of the open questions of the role of phytohormones in plant–microbe interaction. Since *P. indica* colonizes the roots of many host plants, similarities and differences between different host species can be investigated, which ultimately is also relevant for agriculture. Agricultural productivity suffers a heavy loss due to plant pathogens, insect pests and various abiotic stresses. Many endophytic fungi like *P. indica* are able to produce antimicrobial compounds, plant growth hormones and various agrochemical bioactive metabolites and play an important role in plant growth promotion, higher seed yield and plants resistance to various biotic, abiotic stresses, and diseases. Biotechnological applications of the endophytic fungi could be an ideal strategy for sustainable agriculture (Oelmüller et al., 2009). Moreover, endophytic fungi can be applied for advancement of chemical synthesis using genetic engineering tools for the generation of new organisms. Further, growing endophytes in large scale, modifying culture conditions like changing pH, changing growth media and supplying some stimulants might help in getting better production of the particular bioactive compound for agricultural purpose (Varma et al., 2012; Rai et al., 2014).

Chemical mediators released by microbes into the rhizosphere may be crucial players in the rhizosphere and could stabilize symbiotic interactions. Very little is known about chemical compounds which participate in the communication of the community members in the ecosystem. Recently, it was demonstrated that *P. indica* releases cellotriose in nmol concentrations which is perceived by roots and activates a surveillance system that informs the host about the integrity of its cell wall (Johnson et al., 2018). The role of phytohormones in the plant response to such chemical mediators need to be investigated. Cellotriose is one of million chemical mediators which are synthesized and released by microorganisms in extreme low concentrations to facilitate communication among community members. Phytohormones also play important roles in shaping the rhizosphere community by participating in transferring information systemically from the roots to the shoots, but also by transferring information to neighboring plants. Again, the interaction of *P. indica* with *Arabidopsis* roots served as a model system to demonstrate that threat induced by *Alternaria brassicae* in one leaf of a plant induces systemic JA responses in the entire infected plants including the roots. This information travels to neighboring plants via a *P. indica* hyphal network and activates ABA responses (Vahabi et al., 2018). In the context of the ideas by Stec et al. (2016) who proposed that the fluctuation in the ABA levels may work as an alert system that calculates the ratio between costs and incomes for an individual plant, a broader concept for the entire plant/microbe community in an ecosystem could be developed. Well-established model systems also demonstrate that phytohormone-based threat information applied to one plant travels to neighboring plants via mycelial hyphal networks which connect different plants and even plant species in an ecosystem. Threat information about herbivore attacks or pathogen infections can also be distributed in a plant community by volatiles, which induces appropriate phytohormone-based defense responses in non-threatened neighboring plants (see references in Hettenhausen et al., 2017; Vahabi et al., 2018). These examples demonstrate that phytohormones play important roles in shaping community structures. A better understanding of the role of phytohormones in rhizosphere communities will contribute substantially to improve agricultural applications.

## Author Contributions

WZ and RO conceived the topic and outline and critically revised the manuscript. LX prepared the manuscript draft. CW contributed critical components to the draft. All authors reviewed the manuscript.

## Conflict of Interest Statement

The authors declare that the research was conducted in the absence of any commercial or financial relationships that could be construed as a potential conflict of interest.

## ABBREVIATIONS

ABA: abscisic acid
ACC: 1-aminocyclopropane-1-carboxylic acid
AM: arbuscular mycorrhiza
AMF: arbuscular mycorrhizal fungus
CK: cytokinin
ET: ethylene
GA: gibberellin
IAA: indole-3-acetic acid
JA: jasmonic acid
JA-Ile: JA-isoleucine
MAMPs: microbe-associated molecular patterns
MTI: MAMP-triggered immunity
OPDA: 12-oxo-phytodienoic acid
SA: salicylic acid
